# High-Throughput, High-Quality: Benchmarking GNINA and AutoDock Vina for Precision Virtual Screening Workflow †

**DOI:** 10.3390/molecules30163361

**Published:** 2025-08-13

**Authors:** Rocco Buccheri, Antonio Rescifina

**Affiliations:** Department of Drug and Health Sciences, University of Catania, Viale A. Doria 6, 95125 Catania, Italy; rocco.buccheri@unict.it

**Keywords:** HTVS, molecular docking, virtual screening, AutoDock, Vina, GNINA

## Abstract

Drug discovery is an intricate and resource-intensive process in which computational approaches, such as molecular docking, are essential, particularly in the early stages, to identify potential hits. However, docking still has many drawbacks, including problems in managing protein flexibility and the reliability of scoring functions. In this paper, we systematically compared the performance of AutoDock Vina, one of the most widely used open-source docking tools, with GNINA. This advanced evolution integrates convolutional neural networks (CNNs) for pose scoring. The comparison was conducted on ten heterogeneous protein targets, including metalloenzymes, kinases, and G-protein-coupled receptors (GPCRs). With the ability to accurately replicate binding poses and their energy values, GNINA showed outstanding performance in both virtual screening (VS) of active ligands and re-docking steps of co-crystallized ligands. GNINA’s enhanced ability to accurately distinguish between true positives and false positives—a specificity not found with AutoDock Vina—is confirmed by ROC curves and Enrichment Factor (EF) results. Therefore, we propose an integrated GNINA-based workflow that can significantly enhance the quality and reliability of docking results, providing a valuable tool for optimizing the initial stages of drug discovery.

## 1. Introduction

Drug discovery is a complex and expensive process aimed at identifying and developing new drugs, which has seen an increasing integration of computational methodologies in recent decades. These methodologies have become crucial components in many phases of drug discovery programs, from identifying “hits” to optimizing “leads” [1]. Among these, molecular docking has emerged as a fundamental and widely used computational tool [2,3,4].

Currently, molecular docking is an established and popular in silico tool for identifying new compounds with therapeutic potential. It is primarily used in the early stages of drug discovery due to its cost-effectiveness and potential to identify novel chemotype as well as provide mechanistic insights into ligand–protein interactions [2,3]. With the rapid improvement of computational platforms and the substantial increase in structural, chemical, and biological data available for an expanding number of therapeutic targets, in silico approaches, such as docking, have undergone significant growth. Molecular docking has become a crucial component of computational procedures employed in modern drug discovery. Structure-based virtual screening (VS) techniques, which often employ docking, enable the screening of digital libraries comprising millions of compounds within affordable timescales, thereby reducing the initial costs of hit identification and increasing the likelihood of discovering promising drug candidates. Automated workflows integrating docking have been developed for screening large libraries of compounds and targets. The advancement of high-performance computing and Graphics Processing Units (GPUs) has enabled large-scale screening to become possible [2,3,5]. Although initially used as a stand-alone method, docking is now predominantly integrated into workflows that combine different computational methodologies, such as ligand-based approaches, molecular dynamics (MD), binding free energy calculations, and artificial intelligence (AI). This integration aims to overcome some of the inherent limitations of docking and better leverage information from various sources, typically resulting in improved predictive performance in terms of hit rates [2].

Despite its widespread use and considerable progress, molecular docking has inherent limitations that limit its predictive accuracy. The two most relevant and widely recognized limitations of molecular docking are the sampling of conformations and the accuracy of scoring functions. Docking involves finding the most favorable reciprocal configurations, known as “poses,” between a ligand and a protein target. However, sampling all possible conformations of the ligand and receptor is often restricted. Additionally, the target protein is frequently treated as a rigid body during docking for computational reasons, which is a significant limitation. Proteins are dynamic systems with some flexibility, which can be affected by ligand binding, known as “induced fit” effects. Approaches to address protein flexibility, such as utilizing multiple protein structures or integrating with MD simulations, are more complex and require greater computational resources [2,3]. The scoring function is a component of docking that quantifies the interaction between the ligand and the target protein, evaluating the goodness-of-fit of a given pose or ranking different ligands. To achieve the computational efficiency required for screening many compounds, scoring functions are often simplified and based on approximate models of protein–ligand interactions. This simplified modeling is a substantial limitation, as approximate scoring functions very rarely correlate well with experimental binding affinities. Imperfections in the scoring functions remain a significant limiting factor in docking. They lead to inaccuracies in the ranking of predicted poses and poor performance in predicting binding free energy. The need to improve predictive accuracy has led to the development of several strategies to mitigate the limitations of individual scoring functions, such as consensus scoring, which combines the results of several scoring functions, and rescoring methods based on different techniques [1,2,3,4].

In the context of rational drug development, molecular docking represents one of the benchmark computational strategies, particularly in the early stages of VS. The use of in silico screening techniques on libraries containing thousands of compounds enables the identification of potential ligands active toward a protein target, resulting in significant time and cost savings compared to traditional in vitro biological assays, which would be impractical on such a large scale. Optimizing computational workflows to ensure a high hit rate as early as the preliminary stage of VS is crucial. Improving the quality of results in these early stages can, in fact, significantly increase the probability of success in the later stages of pharmaceutical development.

AutoDock Vina (often abbreviated just as Vina) is the most widely used and most integrated molecular docking algorithm within molecular docking software. Trott and Olson developed Vina at the Scripps Research Institute in California as an alternative to its predecessor, AutoDock 4. Key features that have contributed to Vina’s widespread adoption include its speed, improved accuracy over AutoDock 4, and free availability under an open-source license. Its high computational efficiency and ease of use led to very rapid uptake in the docking community, well evidenced by the high number of citations in the original article. AutoDock Vina is considered one of the fastest and most widely used open-source programs for molecular docking [6,7]. Despite its undoubted qualities and broad adoption, Vina has limitations. It has difficulties with increasingly flexible ligands, and its empirical scoring function may have a size-related bias [8,9]. GNINA represents a significant evolution in the field of molecular docking, building on Vina and its fork, Smina. The main innovation of GNINA is the integration of convolutional neural networks (CNNs) for scoring protein–ligand poses. In its typical workflow, GNINA samples ligand poses using Markov Chain Monte Carlo (MCMC) sampling, initially driven by AutoDock Vina’s empirical scoring function. Subsequently, GNINA uses the CNN-based scoring function to “rescore” and rank the poses obtained from MCMC sampling. Unlike empirical or knowledge-based scoring functions, which often assume a linear relationship between structural features and binding affinity, CNNs can model nonlinear relationships and potentially interpret molecular interactions in a more sophisticated way [7,10,11,12,13].

GNINA and Vina were selected for comparison due to key attributes that are strategically important for maximizing the effectiveness of molecular docking in VS. First, both algorithms exhibit a plug-and-play design, meaning they require minimal setup and can be readily installed and operated without extensive configuration. Second, each provides robust quantitative predictions of ligand–receptor binding affinities; Vina delivers estimates of binding free energy, while GNINA predicts binding affinity via p*K* values, enabling direct and meaningful comparison of docking results across platforms. Finally, both tools are open-source, ensuring broad accessibility and allowing researchers to integrate, adapt, and reproduce results with ease.

## 2. Results and Discussion

### 2.1. Energy Values and Conversions

Evaluations of the predicted energy from molecular docking were treated differently for the two algorithms analyzed because they yield different outputs. As for Vina, this is reflected in the output values of predicted energy, expressed in terms of the free energy of binding (Δ*G*), which is calculated in kcal/mol. The Δ*G* value was then converted to the corresponding binding affinity constant (*K*) value using the thermodynamic relationship reported in Equation (1).

*K* = e^(Δ*G*/RT)^ (1)

Equation (1) was solved at a temperature of 310 K, with a gas constant R of 0.0019872036 kcal/(K × mol). Subsequently, the *K* value was transformed into its negative logarithm, p*K*.

GNINA returns several output metrics related to CNN assessment, namely: CNN score, CNN affinity, and CNN_VS. The CNN score is an assessment of the goodness of the generated pose, ranging from 0 to 1, where a score of 1 indicates a pose of higher reliability. The CNN affinity is an expected bond affinity and is expressed in p*K*. Finally, the CNN_VS is the product of the CNN score and CNN affinity and is used to rank compounds in VS, having been found effective in retrospective evaluations [7,10,11,12,13].

### 2.2. Target Validation

The protein target validation phase involved identifying and selecting the most suitable protein model for use in the subsequent VS steps. Only protein models that met the following criteria were considered: (i) presence of a co-crystallized ligand; (ii) availability of an experimental binding affinity value (*K*_i_ or *K*_d_); and (iii) crystallographic resolution of less than 3 Å to ensure an adequate level of structural detail.

The selected proteins represent a diverse and biologically relevant set spanning key therapeutic areas such as neurodegeneration, cancer, metabolism, and immune signaling. They include various enzyme classes (hydrolases, kinases, lyases, deacetylases), GPCRs, and chaperones, providing structural and functional variety to test docking workflows rigorously. Well-characterized 3D structures and experimental affinity data are available for accurate benchmarking. This mix of established and emerging drug targets ensures broad applicability. In Table S1, we reported the grouping by enzyme classification to aid interpretation of docking results by linking performance to biochemical mechanisms, enhancing the robustness and biological relevance of the study.

For each selected model, the docking algorithm’s ability to faithfully reproduce the pose of the co-crystallized ligand was evaluated by calculating the Root Mean Square Deviation (RMSD) as well as its accuracy in estimating the binding affinity by comparing the predicted inhibition constant with the experimentally determined value. In cases where the model had multiple co-crystallized domains, the analysis was conducted on each domain individually, and the one that showed the best performance in terms of structural and energetic accuracy was finally selected. Preliminary studies were performed using GNINA to select the PDB file and ultimately the domain to be used. The choice of GNINA at the PDB file selection stage was based on the fact that the CNN score calculated by the algorithm allows a distinction between receptors with high-quality binding sites and those with poor-quality binding sites. Therefore, receptors with the highest possible CNN score, but never below the threshold of 0.90 recommended in the literature, were selected. In the case of multiple domains present, the protein domain with the highest CNN score was selected [14]. The domains chosen and the CNN scores calculated for each target are shown in Table 1.

All selected protein targets were subjected to docking simulations using both Vina and GNINA to compare their performance directly. The results, presented in Table 2, demonstrate a significant performance improvement achieved with GNINA compared to Vina, in both structural and predictive accuracy.

**Figure 1:**
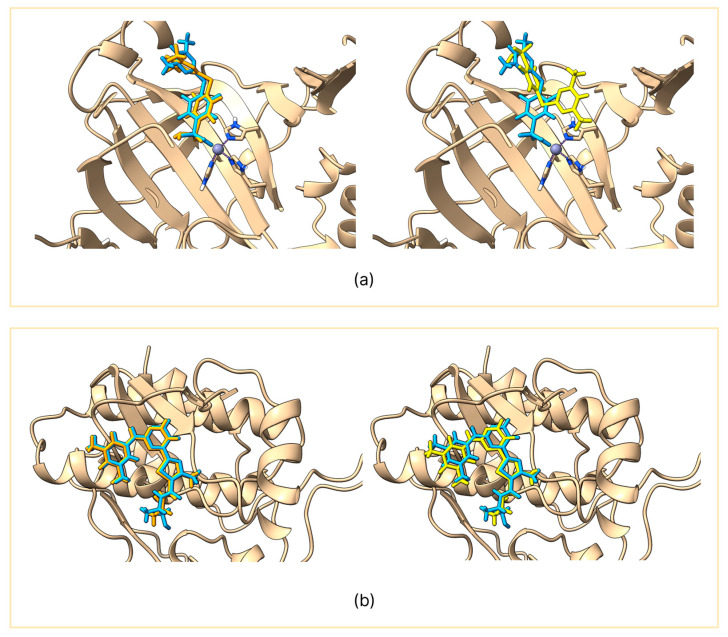
Comparison between the co-crystallized ligand (light blue) and the reproduced pose using GNINA (orange) on the left panel and AutoDock Vina (yellow) on the right panel. The figure shows carbonic anhydrase II (**a**) and SYK kinase (**b**).

GNINA produced RMSD values consistently below 2 Å for the predicted pose, indicating an excellent ability to reproduce the conformation of the co-crystallized ligand [15]. In contrast, Vina showed greater variability: in only 4 out of 10 targets, the RMSD value was less than 2 Å, while in the remaining cases it exceeded 3 Å, showing limited reliability in reproducing the co-crystallized pose. The variability in the redocking of Vina and the stability of GNINA can be observed in the two representative examples shown in Figure 1; the remaining comparisons are presented in Figures S1 and S2.

Similarly, prediction of binding affinity (expressed as p*K*) was also more accurate with GNINA in all targets examined, except in the case of Cyclin-dependent kinase 2, for which both algorithms returned overlapping values.

**Figure 2:**
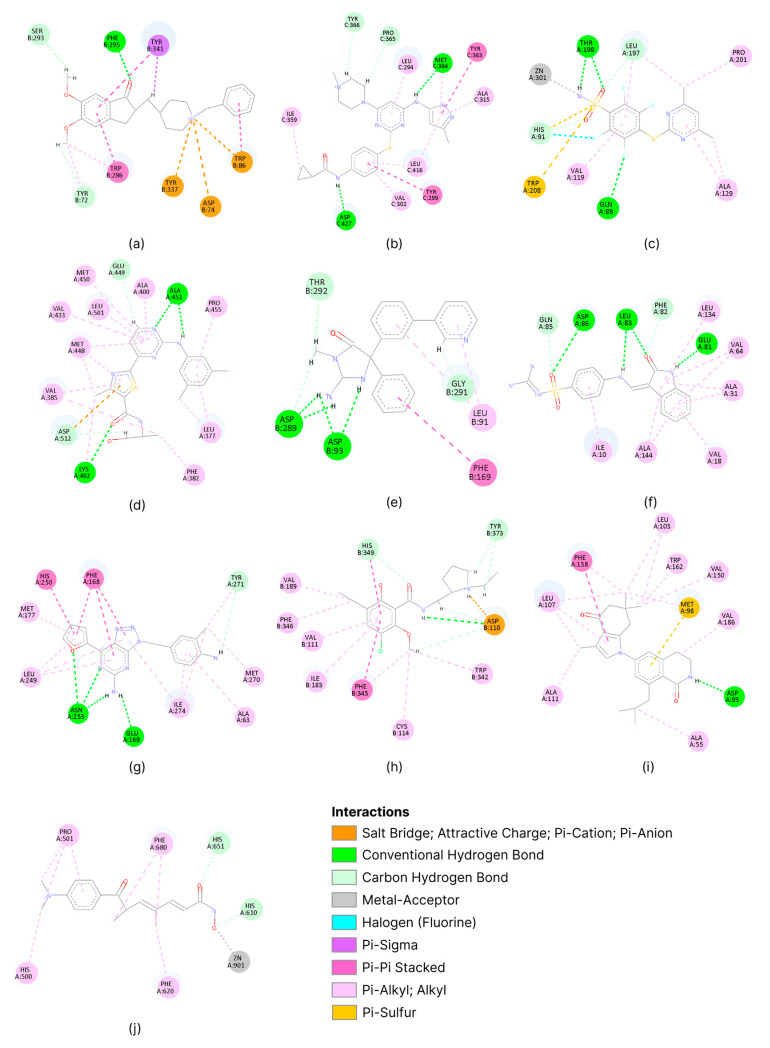
Visual analysis of the physiochemical characteristics of binding sites for acetylcholinesterase (**a**), tyrosine-protein kinase ABL2 (**b**), carbonic anhydrase II (**c**), SYK kinase (**d**), beta-secretase 1 (**e**), cyclin-dependent kinase 2 (**f**), adenosine A2a receptor (**g**), dopamine D3 receptor (**h**), HSP90α (**i**), and HDAC 6 (**j**). Images were generated via BIOVIA Discovery Studio Visualizer (v. 25.1.0.24284).

To understand the different physicochemical properties of the binding sites chosen, the interactions between the co-crystallized ligand and target protein were slightly analyzed (Figure 2).

The selected targets show a marked heterogeneity in terms of the physicochemical properties of their binding sites. The qualitative analysis conducted highlighted the presence of highly polar sites (acetylcholinesterase, beta-secretase 1, and carbonic anhydrase II), sites with balanced polarity-hydrophobic properties (tyrosine-protein kinase ABL2, cyclin-dependent kinase 2, adenosine A2a receptor, and HDAC 6), and predominantly hydrophobic sites (SYK kinase, dopamine D3 receptor, and HSP90α). Furthermore, the set includes both metalloproteins (carbonic anhydrase II and HDAC 6) and proteins without metal cofactors.

### 2.3. Virtual Screening Analysis

VS analyses were conducted using sets of ligands for which experimental affinity values (*K*_i_ or *K*_d_) were available. The criterion adopted for selecting candidate molecules (“hits”) was based on the p*K* value estimated by the docking algorithm. Specifically, a selective filter was applied, considering only molecules with a p*K* ≥ 6.3, a threshold that corresponds to a binding affinity of approximately 500 nM, a value commonly accepted in literature as indicative of good biological activity [16]. At the end of the filtering process, the predictive validity of the system was assessed by comparing the docking results with available experimental data. All molecules with experimental *K*_i_/*K*_d_ values less than 1000 nM were considered effectively active. Based on this comparison, the percentage success rate of VS was calculated according to the formula reported in Equation (2).

(2) $$success\;rate\;(\%)=\frac{number\;of\;experimentally\;active\;hits}{total\;number\;of\;hits\;with\;pK\geq 6.3}\times 100$$

This approach enables a quantitative evaluation of the algorithm’s ability to identify truly active molecules, providing an objective measure of the effectiveness of the screening protocol employed. As in the validation phase, the expected activity value considered in the VS analyses was CNN_VS for the GNINA algorithm and the p*K* value derived from the Vina Δ*G* transformation.

The data shown in Table 3 indicate that, in most cases, the use of GNINA resulted in a higher success rate percentage (SR%) compared with Vina. For 8 out of 10 targets analyzed, GNINA showed a greater ability to identify experimentally active ligands from the screening sets. A particularly significant example is Carbonic anhydrase II, for which GNINA identified 149 hits, 146 (98%) of which were active, compared with only two hits obtained from Vina, of which only one was active.

However, the difference was not marked in all cases. For the SYK kinase target, for example, Vina achieved a hit rate of 85%, slightly higher than that of GNINA (83%). Furthermore, for the Dopamine D3 receptor, both algorithms achieved essentially equivalent results, with a high predictive accuracy of 97%.

### 2.4. ROC Curves and Enrichment Factors Analysis

To statistically evaluate the ability of the two docking methods compared and obtain quantitative estimates of their sensitivity in distinguishing between active and inactive compounds, Receiver Operating Characteristic (ROC) curves with their Area Under the Curve (AUC) and Enrichment Factors (EF) were calculated.

ROC curves are an established graphical and analytical tool. In the context of VS, an ROC curve is constructed by representing the true positive fraction (TPF) on the *y*-axis versus the false positive fraction (FPF) on the *x*-axis. Each point on the curve represents a TPF/FPF pair corresponding to a specific fraction of the molecular dataset classified according to the scoring function scores. A scoring function that can perfectly discriminate between active and inactive compounds (without overlapping score distributions) would have an ROC curve that passes through the upper left corner of the graph (TPF = 1, FPF = 0), indicating perfect sensitivity and specificity. In contrast, a scoring function that has no discriminatory power would produce a curve coincident with the 45° diagonal, corresponding to random selection (AUC = 0.5). The Area Under the ROC Curve (AUC) is a scalar value that summarizes the overall performance of a VS. An AUC value closer to 1 indicates a high discrimination ability of the scoring function in distinguishing between active and inactive compounds over the entire data set. AUC > 0.5 suggests that the prediction ability is better than a random distribution model. Thus, AUC corresponds to the probability of correctly classifying a random pair of active ligand and decoy [17,18,19,20,21].

The EF is used to evaluate the ability of a scoring function or VS method to enrich a subset of selected molecules (typically those with the best scores) in active compounds compared to a random selection from the entire data set. In practical terms, EF at a given percentage X% indicates how many times more active compounds are recovered in the top X% of the library than would be expected from a random selection of the same percentage of compounds. Performance evaluation often focuses on EF at low percentages of the ranked database, since the goal of VS is to quickly identify a small fraction of promising compounds for experimental testing. For example, an EF at 1% (EF1%) equal to 10 means that 10 times more active compounds are found in the top 1% of compounds ranked by the scoring function than in a random selection of 1% of compounds from the entire data set [18,19,20,21,22].

**Figure 3:**
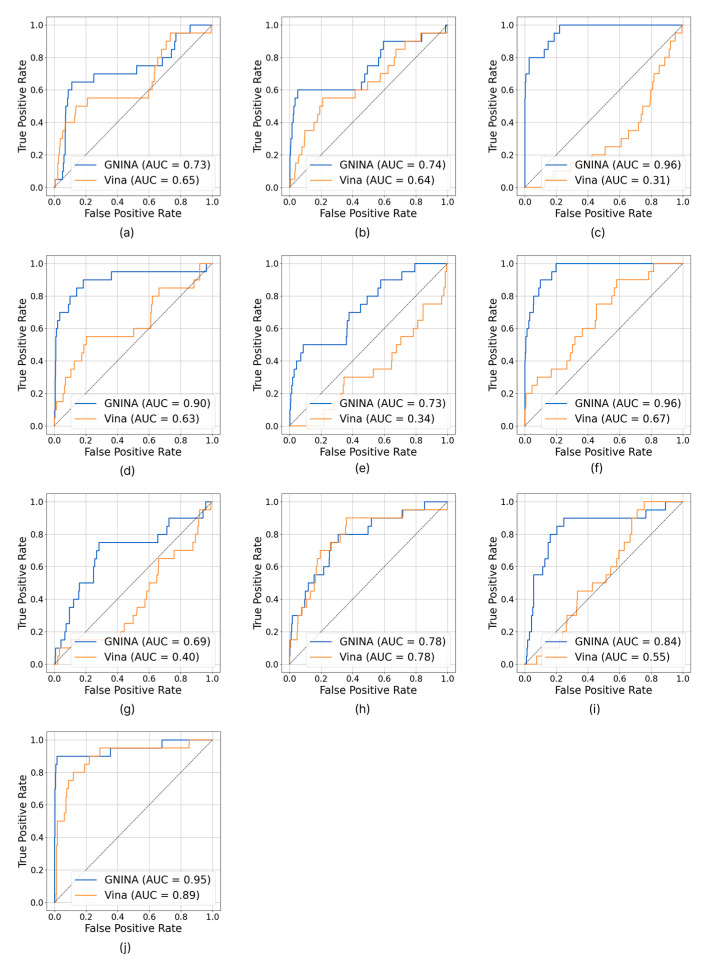
ROC curves and calculated AUC of acetylcholinesterase (**a**), tyrosine-protein kinase ABL2 (**b**), carbonic anhydrase II (**c**), SYK kinase (**d**), beta-secretase 1 (**e**), cyclin-dependent kinase 2 (**f**), adenosine A2a receptor (**g**), dopamine D3 receptor (**h**), HSP90α (**i**), and HDAC 6 (**j**).

Analysis of the ROC curves (Figure 3) clearly shows that GNINA outperforms Vina for all targets considered, except for the dopamine D3 receptor, for which the two methods exhibit overlapping performance. It is particularly relevant to note that GNINA achieved AUC values above 0.70 in almost all cases, never approaching the random classification threshold (AUC ≈ 0.50). In contrast, Vina exceeded the threshold of AUC > 0.5 in only six protein targets.

Analysis of EFs (Table 4 and Figure S3), assessed at the thresholds of 1%, 5%, and 10%, also confirmed the superiority of GNINA over Vina. Out of a total of 30 comparisons, GNINA showed better performance in 25 cases, and the two algorithms showed the same performance in four cases. Vina performed better than GNINA in only one analysis. Only in three circumstances—SYK kinase at EF1%, HSP90α at EF1%, and dopamine D3 receptor at EF10%—does the performance of the two tools appear equivalent.

Furthermore, the physicochemical heterogeneity of the target protein set suggests that the proposed workflow is robust and potentially generalizable to different application contexts. The results obtained indicate that GNINA maintains high performance in various structural and functional environments. We therefore believe that the CNN-based approach adopted by GNINA may benefit from the ability to capture more complex molecular interactions, such as hydrogen bonding patterns, hydrophobic complementarity, or coordination with metal ions, compared to empirical scoring functions such as the one implemented in AutoDock Vina.

We want to emphasize that, as in any VS study, the generation of decoys involves a certain degree of intrinsic uncertainty. Although the decoys used were selected to be structurally similar to the active ones, without experimental evidence of activity, it is not possible to completely exclude that some of them may possess a residual affinity for the target, especially in the case of proteins with promiscuous binding sites. We acknowledge this methodological limitation and believe it is essential to make it explicit to ensure greater transparency in the interpretation of results related to enrichment metrics and ROC analysis.

### 2.5. Proposed GNINA Complete Workflow

**Figure 4:**
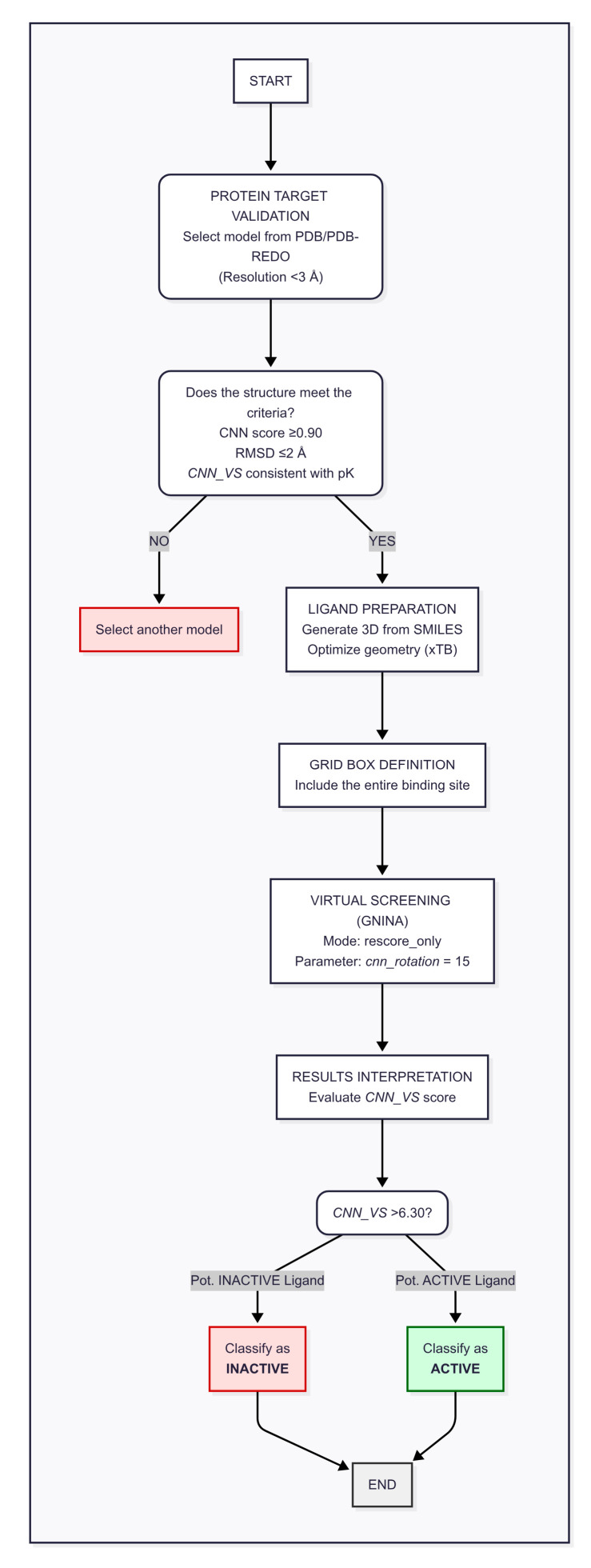
Block diagram workflow.

Based on the results obtained, we propose the following workflow as an operational recommendation for conducting molecular docking studies utilizing GNINA as the docking algorithm (Figure 4). This workflow has been validated specifically in the context of co-crystallized ligands and is ideally applied under such conditions to ensure reliability.

The essential initial step involves thorough structural validation of the protein target. It is advisable to select crystallographic models with resolutions better than 3 Å, prioritizing the most suitable structures available from the RCSB PDB and PDB-REDO databases. Selection criteria should be guided by crystallographic refinement parameters and model quality metrics reported by PDB-REDO. The validity of the target structure must be confirmed according to the following criteria: (i) CNN score ≥ 0.90, indicating a well-defined binding site compatible with the GNINA predictive model; (ii) RMSD ≤ 2 Å, demonstrating the algorithm’s capacity to reproduce the pose of the co-crystallized ligand accurately; and (iii) CNN_VS value consistent with the experimental affinity p*K*, where such data (e.g., *K*_i_ or IC_50_) are available.

In cases where experimental affinity data are unavailable, criterion (iii) may be omitted. When multiple protein models are accessible, priority should be given to those with known experimental affinity values to enable more robust system validation.

Following target validation, three-dimensional ligand structures should be generated from SMILES codes. Initial conversion can be achieved using Open Babel, followed by geometric optimization at a semi-empirical level, such as with xTB, to obtain conformationally accurate ligand geometries.

The simulation grid must be carefully defined to comprehensively encompass the binding site, avoiding overly restrictive boundaries that might artificially bias ligand–protein interactions. It is recommended to maintain a sufficient margin between the active site and the outer protein surface to ensure realistic docking conditions.

Virtual screening should be conducted in rescore mode, wherein the convolutional neural network (CNN) is applied exclusively during the rescoring phase of the generated poses. This approach effectively balances predictive accuracy and computational efficiency. Our tests identified a cnn_rotation parameter value of 15 as optimal, enabling extensive exploration of conformational space without substantially increasing computational cost.

For result interpretation, the CNN_VS value should be treated as an indicator of predicted affinity p*K*. A threshold of 6.30 is recommended to classify ligands: those with CNN_VS > 6.30 are considered potentially active, while those with CNN_VS < 6.30 are regarded as potentially inactive.

## 3. Materials and Methods

### 3.1. Protein Preparation

Protein structure selection was conducted by evaluating the RCSB PDB Databank (https://www.rcsb.org/, accessed on 18 April 2025) and the PDB-REDO Databank (https://pdb-redo.eu/, accessed on 18 April 2025) structure quality, and the best one was chosen considering crystallographic refinement and model quality parameters shown in the PDB-REDO Databank. We exclusively included crystallographic structures in the dataset, which aligns with the use of PDB-REDO, a pipeline specifically designed to optimize and re-refine X-ray crystallographic data. The organism chosen was Homo sapiens, and the resolution was always less than 3 Å. Each protein model selected (Table 1) was prepared in YASARA software (v. 25.1.13, YASARA Biosciences GmbH, Vienna, Austria). All protein structures were prepared under the assumption of physiological pH conditions (7.4), which is consistent with the standard pH range used in most of the reported activity assays for the proteins investigated in our study.

To account for the correct protonation states of ionizable residues, we used automated p*K*_a_ prediction and protonation tools of YASARA during protein preparation, which combine p*K*_a_ prediction with hydrogen bonding network optimization, activated by the command ‘Clean > All’.

Given that Beta-secretase 1 functions in acidic compartments (optimal activity at pH 4.5), we acknowledge that the protonation state at neutral pH may not fully reflect its active conformer. However, for consistency and comparability across the dataset, we modeled all proteins at physiological pH.

Waters and other non-necessary molecules for docking and the co-crystallized ligand were removed using YASARA software. The protein was saved in PDB file format.

### 3.2. Dataset and 3D Structures Generation

Ligand datasets were downloaded from Binding Database (https://www.bindingdb.org/, accessed on 18 April 2025) using the protein name as a research query. Data were filtered by selecting only Homo sapiens as the target source organism, and ligands with known *K*_i_ or *K*_d_ values were chosen. Duplicate molecules were deleted, merging equivalent rows in DataWarrior (powered by openchemlib, v. 06.00.00) [23] using SMILES code as reference. Salts were also deleted using the largest fragment selection option in DataWarrior. Ligands’ 3D structures were generated and minimized in Open Babel (v. 3.1.1) [24], reflecting physiological pH (7.4) states. Then, 3D structures were optimized at a semi-empirical level using the xTB (extended tight-binding, v. 6.7.1) program package [25]. Geometry optimization was performed in xTB using the Analytical Linearized Poisson–Boltzmann (ALPB) model for water, with charge states specified for each molecule according to physiological pH conditions.

### 3.3. Molecular Docking Analysis

Molecular docking analysis was performed using both the molecular docking program GNINA (v. 1.3) [11] and the AutoDock Vina (v. 1.2.5) [26] docking algorithm. Regarding GNINA, we utilized the “rescore” docking mode setting, which involved 15 ligand pose rotations, and the poses were sorted by CNN score. The protein input format was in PDB file format, and the ligand input format was in SDF file format. AutoDock Vina docking analysis was performed by setting the exhaustiveness parameters to 32 and converting the input files to the PDBQT file format required by Vina.

In both cases, the simulation boxes were built using AutoDock Tools (v. 1.5.7), with the co-crystallized ligand pose serving as the reference. Grid parameters (Table S2) were chosen wide enough not to force the ligand–receptor interaction.

### 3.4. RMSD Calculation

RMSD calculation was performed using the tool DockRMSD (v. 1.0.0) [27]. The co-crystallized ligand was converted into a MOL2 file format through Open Babel, as required by the tool. The docking output poses were merged into a single pose, and the first one was converted into a MOL2 file format. The co-crystallized ligand pose and the first docked one were used as input to calculate RMSD.

### 3.5. Decoys Generation

Decoys were prepared via the LUDe web server [28] from the 20 most active ligands for each target, which constituted the true positives. Of the decoys generated by LUDe, 400 were selected for each target, which constituted the false positives. Maintaining an active-to-decoy ratio of approximately 5% was guided by literature precedent, which indicates that highly imbalanced datasets, characterized by a low proportion of active compounds, can improve the robustness and discriminatory power of VS protocols [29,30]. Decoys are decoy molecules constructed from ligands with known experimental activity toward the protein target, having similar physicochemical properties but different 2D topology. The 3D structures of decoys were prepared as reported in Section 3.2.

### 3.6. ROC Curves and Enrichment Factors

ROC curves and related AUC values were calculated using the roc_curve() and auc() functions of the sklearn.metrics module (scikit-learn v. 1.3.0). A set of 420 compounds was prepared for each protein target, including the 20 most active ligands according to experimental affinity values and 400 decoy molecules. The active ligands represented True Positives, while the 400 decoys represented False Positives. False Positive Rate (FPR) and True Positive Rate (TPR) values were obtained by comparing the binary labels with the predictive scores of each docking method. The AUC was subsequently calculated by trapezoidal numerical integration of the ROC curve. ROC curves were generated using matplotlib (v. 3.7.1), plotting TPR vs. FPR for each method with indication of the respective AUC values in the legends. The diagonal reference line (random classifier) was included for visual comparison of performance.

EFs were calculated at the thresholds of 1%, 5%, and 10% of the total dataset. For each predictive method, the dataset was sorted in descending order of score. The number of active compounds in the top-ranked subset was divided by the expected number of active compounds in a random selection of the same size, according to the formula reported in Equation (3).

(3) $$EF=\frac{\left(\mathrm{N\_a}ctiv\mathrm{e\_t}op/\mathrm{N\_t}op\right)}{\left(\mathrm{N\_a}ctiv\mathrm{e\_t}otals/\mathrm{N\_t}otals\right)}$$

where N_active_top represents the number of active compounds in the selected subset, N_top represents the size of the subset, N_active_totals indicates the total number of active compounds in the dataset, and N_totals indicates the total size of the dataset. Figure S3 was generated with the online Screening Explorer Tool (http://stats.drugdesign.fr/, accessed on 31 May 2025) [31].

### 3.7. Ligand–Protein Interactions Analysis

Ligand–protein interactions were analyzed using BIOVIA Discovery Studio Visualizer (v. 25.1.0.24284, Dassault Systèmes Biovia Corp., San Diego, CA, USA) software. The co-crystallized structure was uploaded in PDB file format, adding all hydrogen atoms through the ‘Chemistry > Hydrogen > Add’ option present in the upper toolbar. The co-crystallized pose was selected and defined as the ligand. Then, the interactions were analyzed using the ‘Show 2D Diagram’ option.

## 4. Conclusions

The present study compared the performance of Vina with that of GNINA, which integrates a CNN-based system used for rescoring poses obtained from molecular docking. Vina was chosen as the comparison algorithm because it is widely used and integrated into standard molecular docking software. Therefore, the primary application we aimed to evaluate is a retrospective analysis of VS. We sought to determine whether the ability of CNNs to interpret molecular interactions provides advantages in the search for active compounds when performing VS of large libraries of molecules.

GNINA showed higher accuracy in reproducing the co-crystallized ligand pose and predicting protein binding affinity compared to Vina. RMSD values consistently less than 2 Å were recorded in all analyses performed with GNINA, while Vina showed greater variability with RMSD values often exceeding 3 Å. In predicting binding affinity, GNINA also returned more accurate values than the experimental values reported in the literature.

In VS analyses, GNINA demonstrated a greater ability to identify experimentally active ligands than Vina, achieving excellent success rates in most cases that were superior to those of Vina. A noteworthy result is that of Carbonic anhydrase II, where GNINA recorded several hits almost exclusively populated by experimentally active ligands. To reinforce these observations, more accurate statistical investigations were conducted by generating a decoy library for each protein target. ROC curves were generated, and respective AUC values—an indicator of the algorithm’s ability to discriminate between active and inactive compounds—were calculated. For GNINA, consistently high AUC values were recorded (AUC > 0.70) and were far from the threshold of AUC ≈ 0.50, which would indicate random classification. Vina results were less homogeneous, with values often below the AUC threshold. EF at different rates of 1%, 5%, and 10% was also evaluated to assess the effectiveness of the methods in recovering active compounds from the library fractions. Again, there were results in favor of GNINA, which consistently outperformed Vina.

In summary, we can state that the use of GNINA CNNs in the rescoring phase of the poses leads to benefits in performance in all phases of VS: from pose reproducibility to prediction of binding affinity and discriminatory ability against active compounds. Therefore, in future computational drug searches, we recommend adopting the workflow for VS discussed in this study, which includes a rigorous target validation phase and the use of the GNINA algorithm for VS of compounds, to maximize its effectiveness and significantly increase the chance of success in identifying promising compounds. The integration of AI-based algorithms allows overcoming the limitations of traditionally employed tools, offering new perspectives for in silico approaches used in drug discovery.

## Figures and Tables

**Table 1:** Selected models of protein targets.

Protein Name	Source Database	PDB ID	Domain	CNN Score
Acetylcholinesterase	PDB-REDO	6O4W	B	0.92
Tyrosine-protein kinase ABL2	RCSB PDB	2XYN	C	0.99
Carbonic anhydrase II	PDB-REDO	4HT2	A	0.90
SYK kinase	PDB-REDO	3EMG	A	0.99
Beta-secretase 1	PDB-REDO	4DJW	B	0.98
Cyclin-dependent kinase 2	RCSB PDB	1KE9	A	0.97
Adenosine A2a receptor	PDB-REDO	5OLH	A	0.96
Dopamine D3 receptor	RCSB PDB	7BVQ	B	0.98
HSP90α	PDB-REDO	4O09	A	0.96
HDAC 6	PDB-REDO	5EDU	B	0.97

**Table 2:** Results derived from the VS performed with GNINA and Vina.

Protein Name	p*K*_exp_	GNINA p*K_pred_*	GNINA RMSD	Vina p*K_pred_*	Vina RMSD
Acetylcholinesterase	8.54–7.42	7.29	1.71	7.61	1.19
Tyrosine-protein kinase ABL2	7.52–7.38	8.47	0.79	5.99	6.54
Carbonic anhydrase II	6.82–6.54	7.73	1.37	4.57	6.78
SYK kinase	8.05	7.85	0.97	6.29	1.04
Beta-secretase 1	6.96	6.83	0.44	5.95	7.31
Cyclin-dependent kinase 2	6.68–5.75	6.52	1.80	6.52	1.96
Adenosine A2a receptor	9.10–8.89	7.60	0.29	6.04	8.33
Dopamine D3 receptor	10.00–9.80	7.56	1.69	4.74	6.23
HSP90α	7.70–7.59	7.75	1.05	8.19	0.95
HDAC 6	9.89–6.30	6.88	1.80	4.79	7.30

The p*K* values predicted by Vina are derived from the conversion of the Δ*G* calculated by Vina, and those predicted by GNINA are nothing but the CNN_VS values calculated by GNINA.

**Table 3:** VS results report.

Protein Name	Input Molecules ^a^	Hits ^b^ (GNINA)	Actives ^c^ (GNINA)	SR% ^d^ GNINA	Hits ^b^ (Vina)	Actives ^c^ (Vina)	SR% ^d^ Vina
Acetylcholinesterase	790	148	90	64%	532	233	44%
Tyrosine-protein kinase ABL2	92	20	11	55%	41	16	39%
Carbonic anhydrase II	502	149	146	98%	2	1	50%
SYK kinase	80	41	34	83%	13	11	85%
Beta-secretase 1	2345	660	574	87%	150	122	81%
Cyclin-dependent kinase 2	1322	726	655	90%	386	285	74%
Adenosine A2a receptor	7001	1630	1426	88%	2839	2129	75%
Dopamine D3 receptor	150	37	36	97%	39	38	97%
HSP90α	637	190	125	63%	299	181	61%
HDAC 6	225	36	34	94%	21	18	86%

^a^ The number of molecules contained in the ligand sets with known activity. ^b^ The number of hits obtained for both algorithms by following the above-mentioned filtering criterion. ^c^ The number of experimentally active ligands present in the hits. ^d^ The percentage of the success rate values.

**Table 4:** EF analysis results report.

Protein Name	GNINA EF1% ^a^	Vina EF1% ^a^	GNINA EF5% ^b^	Vina EF5% ^b^	GNINA EF10% ^c^	Vina EF10% ^c^
Acetylcholinesterase	5.52	5.52	1	6.02	5.52	4.01
Tyrosine-protein kinase ABL2	15	5	10	3	6	2.5
Carbonic anhydrase II	20.75	0	14.53	0	8.1	0
SYK kinase	10	10	12	3	7	3
Beta-secretase 1	12.53	0	7.31	0	4.57	0
Cyclin-dependent kinase 2	20.5	10.25	12.3	4.1	8	3
Adenosine A2a receptor	5.08	0	3.04	2.03	2.54	1.01
Dopamine D3 receptor	15.34	10.22	6.13	3.07	3.58	3.58
HSP90α	0	0	4.01	0	5.51	0
HDAC 6	20.15	0	17.13	10.07	9.07	6.04

^a^ EF calculated at 1%. ^b^ EF calculated at 5%. ^c^ EF calculated at 10%.

## Data Availability

The original data presented in the study are openly available in the HTHQ-GNINA GitHub (https://github.com/, accessed on 31 May 2025) repository at https://github.com/rocco-b/HTHQ-GNINA (accessed on 31 May 2025).

## Supplementary Materials

The following supporting information can be downloaded at: https://www.mdpi.com/article/10.3390/molecules30163361/s1, Figure S1: Comparison between the co-crystallized ligand (light blue) and the reproduced pose using GNINA (orange) on the left panel and AutoDock Vina (yellow) on the right panel. The figure shows acetylcholinesterase (a), tyrosine-protein kinase ABL2 (b), beta-secretase 1 (c), and cyclin-dependent kinase 2 (d); Figure S2: Comparison between the co-crystallized ligand (light blue) and the reproduced pose using GNINA (orange) on the left panel and AutoDock Vina (yellow) on the right panel. The figure shows adenosine A2a receptor (a), dopamine D3 receptor (b), HSP90α (c), and HDAC6 (d); Figure S3: Enrichment curves of acetylcholinesterase (a), tyrosine-protein kinase ABL2 (b), carbonic anhydrase II (c), SYK kinase (d), beta-secretase 1 (e), cyclin-dependent kinase 2 (f), adenosine A2a receptor (g), dopamine D3 receptor (h), HSP90α (i), and HDAC6 (j). Table S1: Enzymatic classification of the protein targets studied, based on the Enzyme Commission (EC) numbering system where applicable, or protein family classification for non-enzymes such as GPCRs and molecular chaperones. Table S2: Grid parameters used in both GNINA and Vina docking analysis. Spacing was always equal to 1. The table shows the grid center coordinates (center x, center y, center z) and the chosen grid points (npts x, npts y, npts z).

## Abbreviations

The following abbreviations are used in this manuscript:

%SR	Success Rate Percentage
AI	Artificial Intelligence
ALPB	Analytical Linearized Poisson-Boltzmann
AUC	Area Under Curve
CNNs	Convolutional Neural Networks
EF	Enrichment Factor
FPF	False Positive Fraction
FPR	False Positive Rate
GPUs	Graphics Processing Units
MCMC	Markov Chain Monte Carlo
MD	Molecular Dynamics
RMSD	Root Mean Square Deviation
ROC	Receiver Operating Characteristic Curve
TPF	True Positive Fraction
TPR	True Positive Rate
VS	Virtual Screening
