# Supplementary material for: High-Throughput, High-Quality: Benchmarking GNINA and AutoDock Vina for Precision Virtual Screening Workflow
Source: Molecules. 2025 Aug 13;30(16):3361. doi: 10.3390/molecules30163361 (PMC12388557; doi:10.3390/molecules30163361)
Supplement: Supplementary file 1 [file molecules-30-03361-s001.zip › molecules-3791594-supplementary.pdf]

molecules

MDPI

Supporting Information

High-Throughput, High-Quality: Benchmarking GNINA and AutoDock Vina for Precision Virtual Screening Workflow †

Rocco Buccheri and Antonio Rescifina *

Department of Drug and Health Sciences, University of Catania, Viale A. Doria 6, 95125 Catania, Italy; rocco.buccheri@unict.it
* Correspondence: antonio.rescifina@unict.it

† In memory of Professor Carmela Spatafora—beloved friend, esteemed colleague, and passionate scientist—on the ninth anniversary of her untimely passing.

Table of Contents

Figure S1. Comparison between the co-crystallized ligand (light blue) and the reproduced pose using GNINA (orange) on the left panel and AutoDock Vina (yellow) on the right panel. The figure shows ace-tylcholinesterase (a), tyrosine-protein kinase ABL2 (b), beta-secretase 1 (c), and cyclin-dependent kinase 2 (d)………….....…S2
Figure S2. Comparison between the co-crystallized ligand (light blue) and the reproduced pose using GNINA (orange) on the left panel and AutoDock Vina (yellow) on the right panel. The figure shows adenosine A2a receptor (a), dopamine D3 receptor (b), HSP90α (c), and HDAC6 (d)…………………………………………...…S3
Figure S3. Enrichment curves of acetylcholinesterase (a), tyrosine-protein kinase ABL2 (b), carbonic anhydrase II (c), SYK kinase (d), beta-secretase 1 (e), cyclin-dependent kinase 2 (f), adenosine A2a receptor (g), dopamine D3 receptor (h), HSP90α (i), and HDAC6 (j)…………………………………………………………...…S4
Table S1. Enzymatic classification of the protein targets studied, based on the Enzyme Commission (EC) numbering system where applicable, or protein family classification for non-enzymes such as GPCRs and molecular chaperones…………………S5
Table S2. Grid parameters used in both GNINA and Vina docking analysis. Spacing was always equal to 1. The table shows the grid center coordinates (center x, center y, center z) and the chosen grid points (npts x, npts y, npts z)…………………………………………………………………………………..... …S6
Shared Files……………………………………………………………………….…S6

 https://doi.org/10.3390/molecules30163361

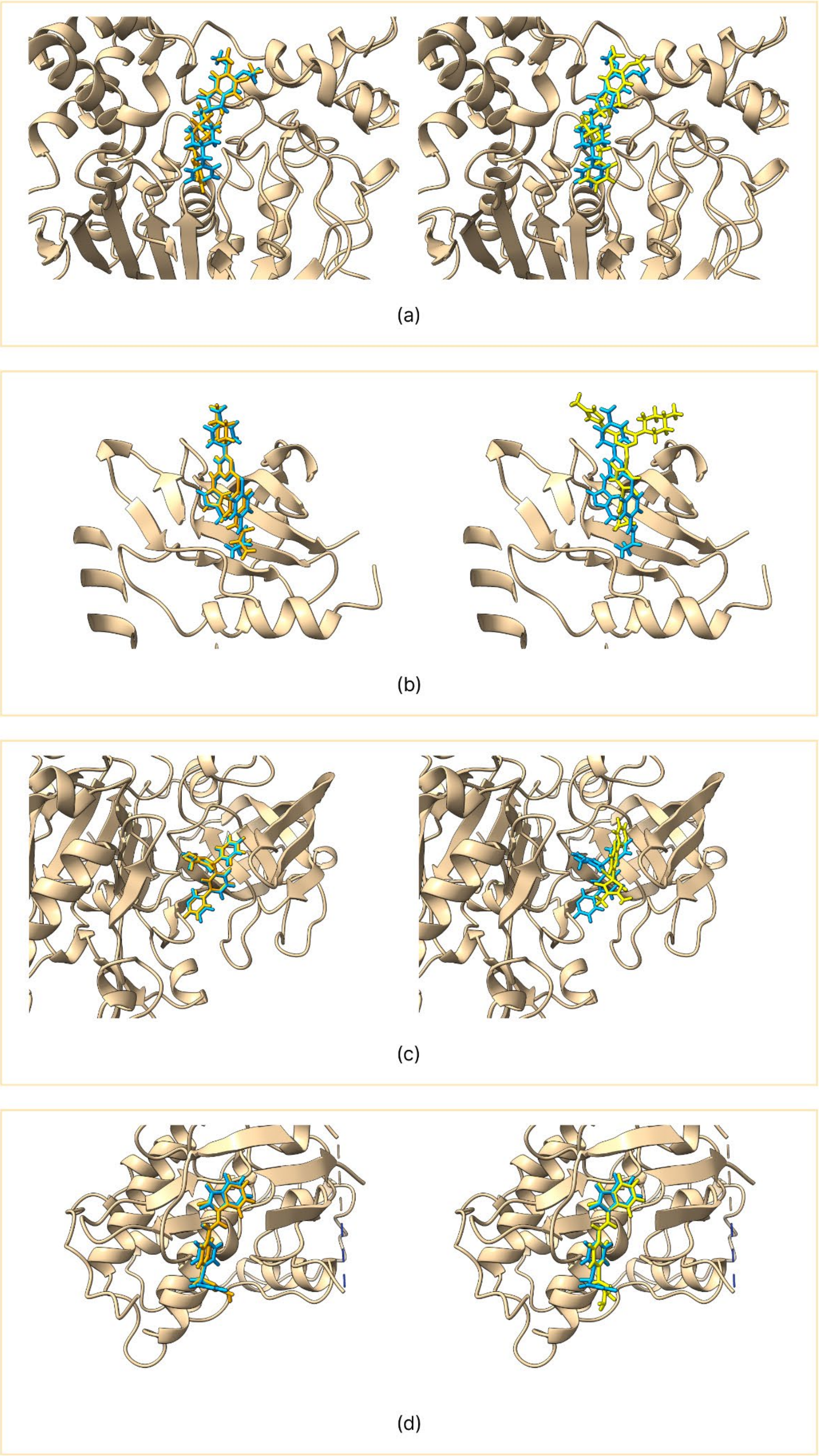

Figure S1. Comparison between the co-crystallized ligand (light blue) and the reproduced pose using GNINA (orange) on the left panel and AutoDock Vina (yellow) on the right panel. The figure shows acetylcholinesterase (a), tyrosine-protein kinase ABL2 (b), beta-secretase 1 (c), and cyclin-dependent kinase 2 (d).

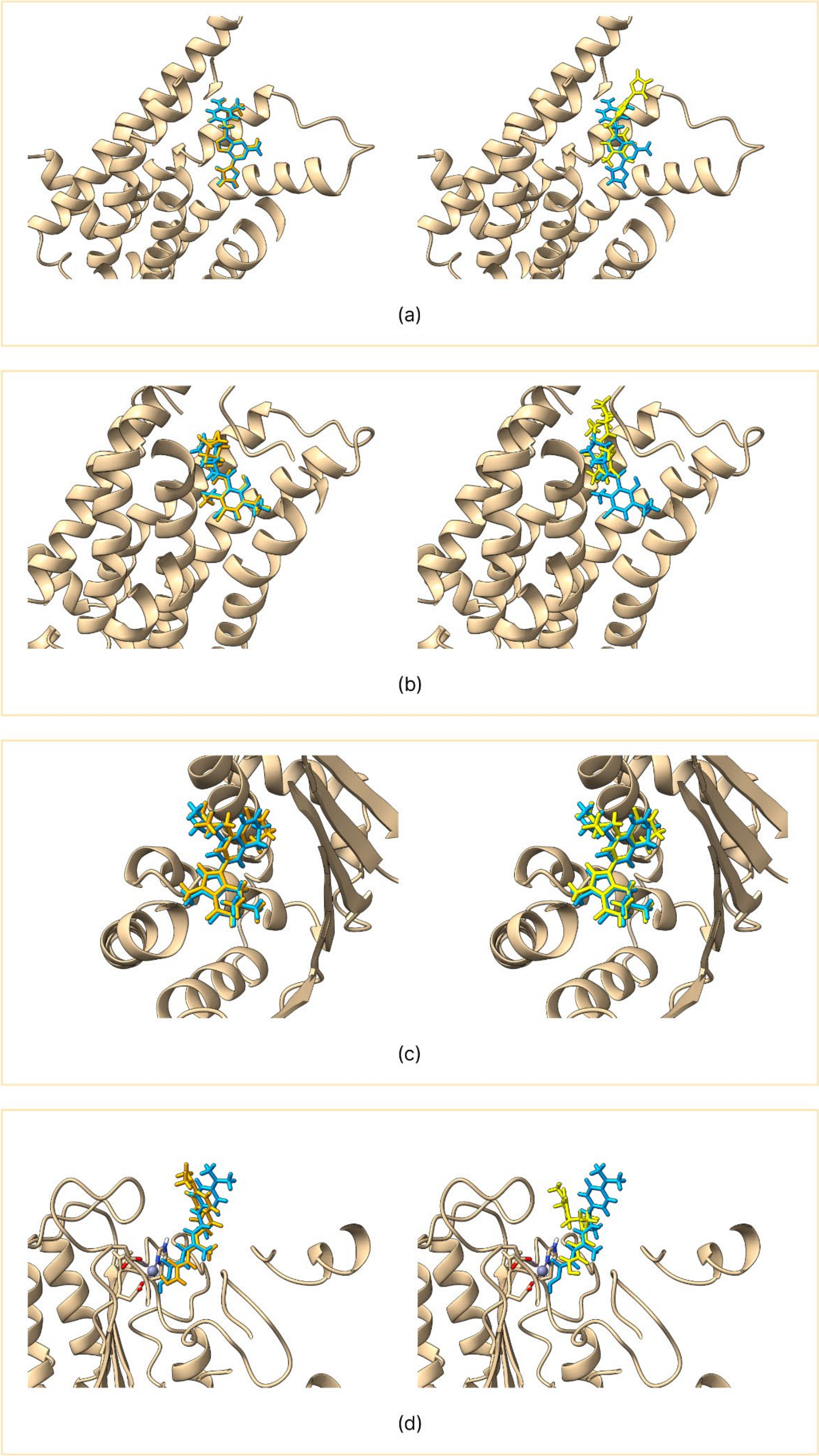

Figure S2. Comparison between the co-crystallized ligand (light blue) and the reproduced pose using GNINA (orange) on the left panel and AutoDock Vina (yellow) on the right panel. The figure shows adenosine A2a receptor (a), dopamine D3 receptor (b), HSP90α (c), and HDAC6 (d).

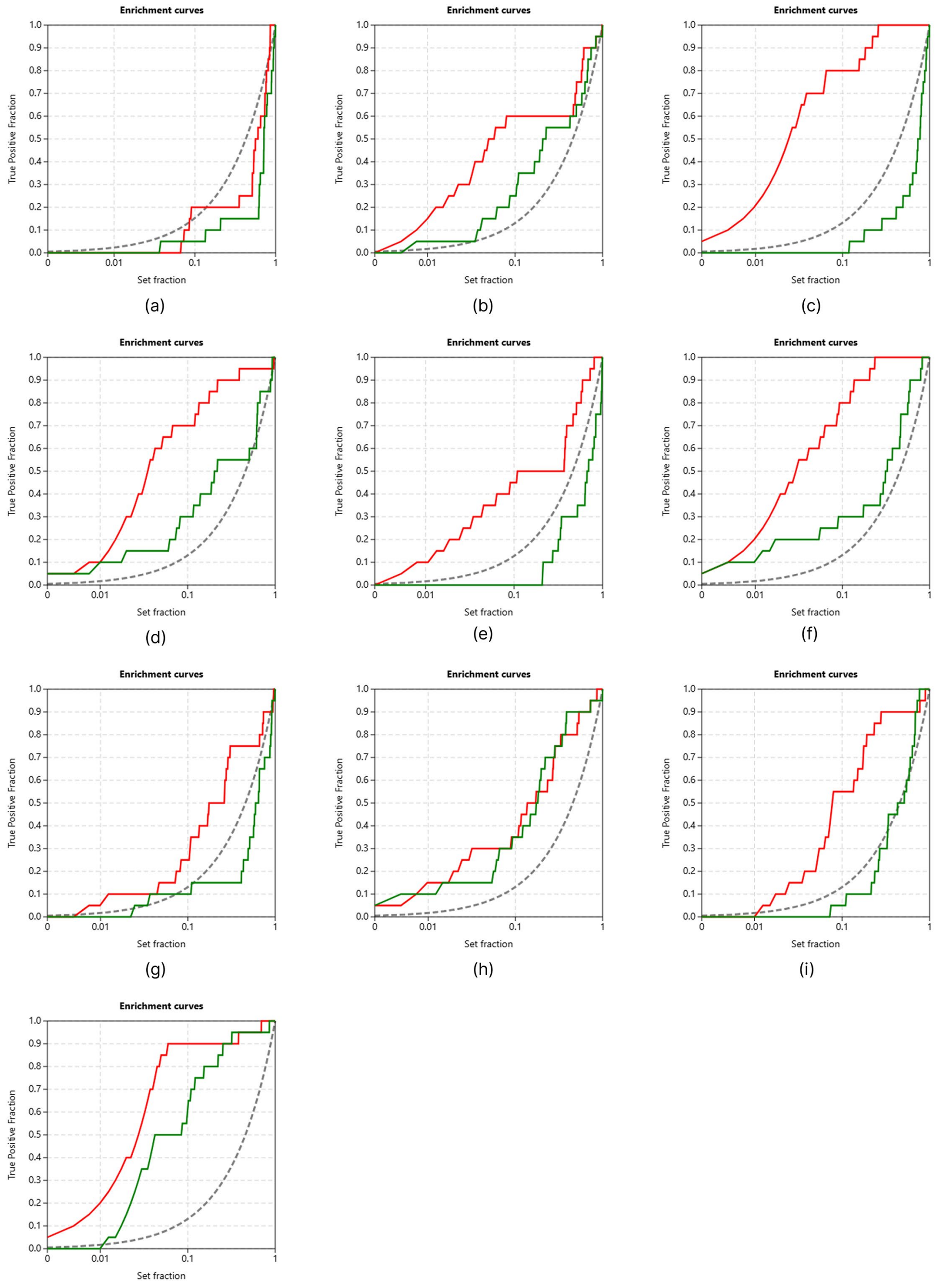

Figure S3. Enrichment curves of acetylcholinesterase (a), tyrosine-protein kinase ABL2 (b), carbonic anhydrase II (c), SYK kinase (d), beta-secretase 1 (e), cyclin-dependent kinase 2 (f), adenosine A2a receptor (g), dopamine D3 receptor (h), HSP90α (i), and HDAC6 (j). Figure was generated with the online Screening Explorer Tool (http://stats.drugdesign.fr/, accessed 05/31/2025) [1].

Table S1. Enzymatic classification of the protein targets studied, based on the Enzyme Commission (EC) numbering system where applicable, or protein family classification for non-enzymes such as GPCRs and molecular chaperones.

Protein Name	EC Number	Enzyme Class/Family	Enzymatic Function/Key Notes
Acetylcholinesterase	3.1.1.7	Hydrolase (Carboxylic-ester hydrolase)	Hydrolyzes acetylcholine to choline and acetate; critical for neurotransmission termination.
Tyrosine-protein kinase ABL2	2.7.10.2	Transferase (Protein-tyrosine kinase)	Non-receptor tyrosine kinase involved in cell signaling and oncogenesis.
Carbonic anhydrase II	4.2.1.1	Lyase (Carbonic anhydrase)	Reversible hydration of CO_2 to bicarbonate; important for pH regulation and CO_2 transport.
SYK kinase	2.7.10.2	Transferase (Protein-tyrosine kinase)	Key mediator in immune receptor signaling pathways in hematopoietic cells.
Beta-secretase 1	3.4.23.46	Hydrolase (Aspartic endopeptidase)	Cleaves amyloid precursor protein; implicated in Alzheimer's disease pathology.
Cyclin-dependent kinase 2	2.7.11.22	Transferase (Protein-serine/threonine kinase)	Regulates cell cycle progression by phosphorylating target proteins.
Adenosine A2a receptor	N/A	G protein-coupled receptor (Class A GPCR)	Mediates physiological responses to adenosine; drug target in cardiovascular and neurological conditions.
Dopamine D3 receptor	N/A	G protein-coupled receptor (Class A GPCR)	Dopamine receptor subtype modulating cognitive and emotional processes; implicated in neuropsychiatric disorders.
HSP90α	N/A	Molecular chaperone (ATPase)	Assists in folding and stabilization of client proteins, especially under stress; essential in cancer biology.
HDAC 6	3.5.1.98	Hydrolase (Deacetylase)	Removes acetyl groups from histone and non-histone proteins; regulates gene expression and cytoskeleton dynamics.

Table S2. Grid parameters used in both GNINA and Vina docking analysis. Spacing was always equal to 1. The table shows the grid center coordinates (**center x**, **center y**, **center z**) and the chosen grid points (**npts x**, **npts y**, **npts z**).

Protein name	Center x	Center y	Center z	npts x	npts y	npts z
Acetylcholinesterase	89.919	94.443	17.303	28	24	20
Tyrosine-protein kinase ABL2	−63.101	10.422	−34.090	20	22	18
Carbonic anhydrase II	−3.826	24.016	−4.778	26	20	24
SYK kinase	17.324	40.930	9.178	22	30	26
Beta-secretase 1	16.915	30.739	57.257	30	24	22
Cyclin-dependent kinase 2	−9.642	48.089	9.126	22	20	24
Adenosine A2a receptor	−21.392	11.064	15.644	22	32	26
Dopamine D3 receptor	3.533	18.093	−15.119	20	26	24
HSP90α	2.893	−7.723	−24.333	22	20	24
HDAC 6	0.053	9.322	5.058	24	22	20

Shared Files

CSV file 1 "experimental_ligands_data.csv". Complete database used in VS reporting SMILES codes of each compound (SMILES), experimental K_i/K_d numeric value expressed in nM (K), the CNN_VS values (CNN_VS), the pK_i derived from Vina output (Vina pK_i) and the corresponding target protein (Target protein). Note that Vina failed to analyze 13 ligands reported as #NUM! values in the Vina pK_i column.

CSV file 2 "decoy_data.csv". Complete database used in VS reporting SMILES codes of each decoy molecule (SMILES), the CNN_VS values (CNN_VS), the pK_i derived from Vina output (Vina pK_i), and the corresponding target protein (Target protein). Note that Vina failed to analyze 172 ligands reported as #NUM! values in the Vina pK_i column.

Python script "ROC_AUC_EF.py". Python script used in ROC-AUC and EFs calculations.

These data are openly available in HTHQ-GNINA GitHub repository at https://github.com/rocco-b/HTHQ-GNINA.

References

1. Empereur-Mot, C.; Zagury, J.-F.; Montes, M., Screening Explorer–An Interactive Tool for the Analysis of Screening Results. *J. Chem. Inf. Model.* **2016**, 56, 2281–2286. https://doi.org/10.1021/acs.jcim.6b00283.
